# Single-shot experiments at the soft X-FEL FERMI using a back-side-illuminated scientific CMOS detector

**DOI:** 10.1107/S1600577521012303

**Published:** 2022-01-01

**Authors:** Cyril Léveillé, Kewin Desjardins, Horia Popescu, Boris Vondungbo, Marcel Hennes, Renaud Delaunay, Emmanuelle Jal, Dario De Angelis, Matteo Pancaldi, Emanuele Pedersoli, Flavio Capotondi, Nicolas Jaouen

**Affiliations:** a Synchrotron SOLEIL, L’Orme des Merisiers, Saint-Aubin, BP48, 91192 Gif-sur-Yvette, France; b Sorbonne Université, CNRS, Laboratoire de Chimie Physique-Matière et Rayonnement, LCPMR, 75005 Paris, France; c Elettra-Sincrotrone Trieste, Basovizza, Trieste 34149, Italy

**Keywords:** CMOS, soft X-ray FEL applications, single-shot experiment, time resolved

## Abstract

The latest Complementary Metal Oxide Semiconductor (CMOS) 2D sensors now rival the performance of state-of-the-art photon detectors for optical application, combining a high-frame-rate speed with a wide dynamic range. While the advent of high-repetition-rate hard X-ray free-electron lasers (FELs) has boosted the development of com­plex large-area fast CCD detectors in the extreme ultraviolet (EUV) and soft X-ray domains, scientists lacked such high-performance 2D detectors, principally due to the very poor efficiency limited by the sensor processing. Recently, a new generation of large back-side-illuminated scientific CMOS sensors (CMOS-BSI) has been developed and commercialized. One of these cost-efficient and com­petitive sensors, the GSENSE400BSI, has been implemented and characterized, and the proof of concept has been carried out at a synchrotron or laser-based X-ray source. In this article, we explore the feasibility of single-shot ultra-fast ex­peri­ments at FEL sources operating in the EUV/soft X-ray regime with an AXIS-SXR camera equipped with the GSENSE400BSI-TVISB sensor. We illustrate the detector capabilities by performing a soft X-ray magnetic scattering experiment at the DiProi end-station of the FERMI FEL. These measurements show the possibility of integrating this camera for collecting single-shot images at the 50 Hz operation mode of FERMI with a cropped image size of 700 × 700 pixels. The efficiency of the sensor at a working photon energy of 58 eV and the linearity over the large FEL intensity have been verified. Moreover, on-the-fly time-resolved single-shot X-ray resonant magnetic scattering imaging from prototype Co/Pt multilayer films has been carried out with a time collection gain of 30 com­pared to the classical start-and-stop acquisition method performed with the conventional CCD-BSI detector available at the end-station.

## Abstract 2

We demonstrate the possibility of using a CMOS 2D sensor for time-resolved pump–probe XUV FEL experiments. We gained a factor of 30 in acquisition time compared to the use of a commercial CCD-BSI.

## 1. Introduction

Nowadays, commercially available Complementary Metal Oxide Semiconductor (CMOS) 2D sensors are largely used for many visible applications or in the X-ray domain coupled with scintillators. The CMOS grade sensor provides a high signal-to-noise ratio with an electronic noise between 1 and 2 e^−^ RMS. The GPIXEL (https://www.gpixel.com/) CMOS GSEN­SE­400BSI is the first large production of this kind of sensor whose manufacturing process is com­patible with soft X-ray applications due to the absence of a microlens, window and to the thin coating applied to the surface. It is based on 2048 × 2048 pixels of 11 µm^2^ with two gains and a combined gain mode to achieve the high dynamic range (HDR). The illumination on the back side (BSI), the thin silicon epitaxial thickness and the entrance window coating layer enable a good efficiency to be obtained over the entire XUV and soft X-ray domain (Desjardins *et al.*, 2020 ▸). The dynamic range can reach 92 dB with a low electronic noise (down to 2 e^−^ RMS) and a relatively large charge full-well capacity (up to 100 ke^−^). In addition, this sensor has a good spatial performance and a com­petitive dark current (∼3 e^−^ s^−1^ pixel^−1^ at −20 °C). The sensor has met with a certain success and it is already integrated by several camera manufacturers for UV and visible applications (https://andor.oxinst.com, https://www.photometrics.com, https://www.ximea.com, https://www.pco.de, http://www.tucsen.com, *etc*.). Recently, two ver­sions have been evaluated at beamlines of Synchrotron SOLEIL (Desjardins *et al.*, 2020 ▸), reporting a sufficient X-ray quantum efficiency and showing the particular inter­est in this CMOS technology. It has been shown that these sensors dramatically increase the full-frame acquisition speed (48 Hz for the standard gain and 24 Hz for the HDR mode) com­pared to the commercial Back-Side-Illuminated Charge-Coupled Device (CCD-BSI) routinely used at soft X-ray facilities. The first utilization of this device opened up perspectives for soft X-ray ptychography (Mille *et al.*, 2022 ▸), soft X-ray scattering techniques (Desjardins *et al.*, 2020 ▸; Marras *et al.*, 2021 ▸) and other applications, such as soft X-ray fluorescence (Staeck *et al.*, 2021 ▸). Another obvious application concerns the implementation of such a class of detectors at ultra-short X-ray sources (laser-based X-ray source or free-electron laser), where the low frame rate of the standard commercially available CCD-BSI (see, for example, Vodungbo *et al.*, 2012 ▸; Wang *et al.*, 2012 ▸) limits the time of data collection. If a more sophisticated CCD-BSI camera can fulfil the requirements of these applications, such as the FastCCD (Denes *et al.*, 2009 ▸) or PnCCD (Strüder *et al.*, 2016 ▸), as well as the large detector development based on DEPFET sensors (Porro *et al.*, 2012 ▸), the commercial CMOS considered here could dramatically decrease the collection time, together with helping with other important criteria for the user, such as low cost, low development or/and experimental integration time needed and a good dynamic with medium signal saturation (up to 80 ke^−^ in HRD mode, *i.e.* 10000 photons of 200 eV) com­pared to readout noise (few electrons, *i.e.* less than 1 photon of 200 eV). In order to illustrate the possibilities offered by this sensor for experiments carried out with a FEL beam, an AXIS-SXR camera supplied by AXIS Photonique (https://www.axis-photon.com/), equipped with a standard GSENSE400BSI-TVISB sensor, has been installed at the DiProI end-station (Capotondi *et al.*, 2013 ▸, 2015 ▸) at the FERMI FEL source. In this article, we describe the setup and the method employed to evaluate the camera performance, particularly in terms of the detector response linearity and its application for time-resolved experiments. We report the first results of FEL single-shot scattering images collected from a prototype magnetic multilayer sample widely studied in recent years (Stamps *et al.*, 1997 ▸; Pfau *et al.*, 2012 ▸; Vodungbo *et al.*, 2012 ▸; Wang *et al.*, 2012 ▸; Willems *et al.*, 2017 ▸). Sorting the collected data on the basis of pulse-deposited energy, we unequivocally observed a loss of the magnetic scattering efficiency before the sample damage threshold, in agreement with previously reported data gathered on the same material both in the EUV and in the soft X-ray regime (Schneider *et al.*, 2020 ▸; Philippi-Kobs *et al.*, 2021 ▸; Wang *et al.*, 2012 ▸; Wu *et al.*, 2016 ▸). Finally, as a potential application of the CMOS-BSI camera in FEL-based time-resolved experiments, a proof-of-principle experiment with the goal of measuring the gain in experimental time with respect to commercially available CCD detectors is given. Multiple ultra-fast demagnetization curves were recorded on a striped-domain Co/Pt sample, continuously varying the arrival time between an IR laser pulse and the FEL, and collecting single-shot scattering frames. Comparing the obtained demagnetization curves with results obtained moving the delay line step-by-step and collecting a diffraction frame in an integrative way using the available end-station CCD-BSI detector, we estimate a gain in the acquisition time by about a factor of 30.

## 2. Materials and methods

**Figure 1:**
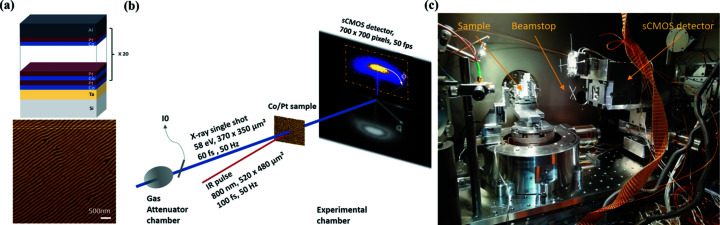
(*a*) Sketch of the multilayer com­position exhibiting out-of-plane magnetic anisotropy. A magnetic domain structure exhibiting aligned stripe domains at remanence is shown by the magnetic force microscope (MFM) image (5 µm × 5 µm) obtained using an in-plane demagnetization procedure. (*b*) Scheme of the pump–probe experiment performed in transmission geometry. (*c*) Photograph of the CMOS setup at the DiProi end-station.

The AXIS-SXR camera is the first vacuum-com­patible camera equipped with a CMOS-BSI sensor available for X-ray applications developed by AXIS Photonique in collaboration with the SOLEIL synchrotron detector group. This first version is equipped with a ‘standard’ GPIXEL CMOS-BSI sensor developed for visible spectrum applications, the GSENSE400BSI-TVSIB. As reported by Desjardins *et al.* (2020 ▸), the sensor has a maximum quantum efficiency (QE) around an energy of 1000 eV and a poor efficiency for lower beam energies (due to the surface anti­reflection protective coating). Compared to the prototype noncoated version, GSENSE400BSI-GP, or the latest version introduced recently by GPIXEL, GSENSE400BSI-Pulser (Harada *et al.*, 2020 ▸; https://www.gpixel.com), the efficiency of GSENSE400BSI-TVSIB is only 20% around 50 eV. Despite these unfavourable detection conditions, the AXIS-SXR camera was installed in the 5 × 10^−7^ mbar vacuum experimental chamber of the DiProi end-station at FERMI for a first proof-of-principle pump–probe XUV experiment [Fig. 1 ▸(*c*)]. The camera was installed on a bidimensional motorized stage that allows alignment of the chip with respect to the FEL beam direction and varying of the sample-to-detector distance in order to optimize the experimental scattering geometry. Moreover, a free-standing Al filter (200 nm thick) was installed in front of the CMOS-BSI chip to protect the detector from saturation induced by IR stray light during the time-resolved experiment. Finally, the direct FEL beam was blocked by a metallic beamstop placed before the sensor on a carousel rotation stage placed on the body case of the detector head. The camera was controlled through the Lima tango (https://www.tango-controls.org) device for a quick and simple integration into the beamline control system.

**Figure 2:**
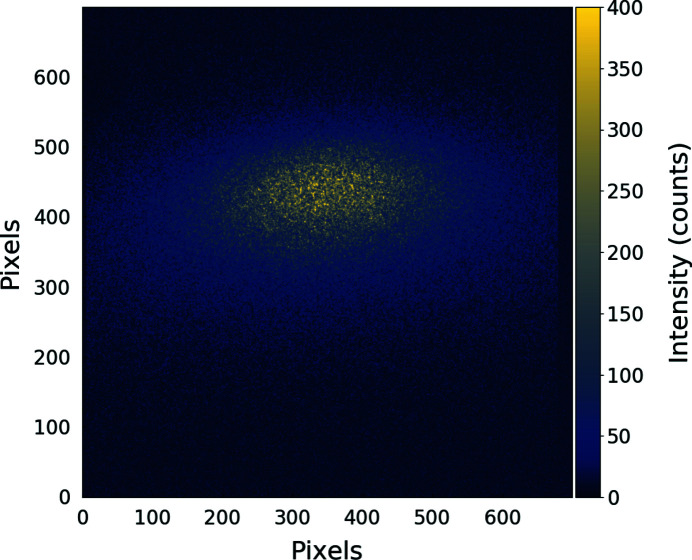
Dark corrected image of a diffraction peak of striped domains in a Co/Pt multilayer recorded using the AXIS-SRW camera with an integration time of 14.5 ms in HDR mode.

In order to test the detector, we investigated the pump–fluence dependence of the ultrafast demagnetization of a Co/Pt multilayer [Fig. 1 ▸(*a*)] sample (Si_3_N_4_/Ta2nm/[Co0.6nmPt0.8nm]20/Al5nm) by X-ray resonant magnetic scattering (XRMS) at the cobalt *M*-edge. Prior to the experiment, a demagnetization procedure with an oscillating decreasing magnetic field, oriented parallel to the film surface, was employed to obtain a magnetic domain structure of well-aligned stripe domains [Fig. 1 ▸(*b*)] (Hellwig *et al.*, 2003 ▸). By tuning the probe wavelength to the magnetically dichroic Co *M*-edges, this grating-like magnetic periodic structure gives rise to magnetic diffraction peaks localized on both sides of the transmitted beam, as shown in Fig. 1 ▸(*b*). The diffraction pattern is recorded by the camera placed downstream in transmission geometry, as shown schematically in Fig. 1 ▸(*b*) and illustrated in the photograph in Fig. 1 ▸(*c*). The camera was 15.5 cm behind the sample and aligned 2.5 cm off with respect to the FEL propagation axis. In order to collect frames synchronized with the 50 Hz repetition rate of the FEL source, we generously cropped an area of 700 × 700 pixels of the acquisition chip. We would like to point out that for a particular purpose, if cropped in 64 × 2048 pixels, one can reached a 713 Hz repetition rate with the sensor used in our experiment. Compared to the usual DiProi CCD-BSI camera (Princeton Instruments, MTE2048B), the same number of pixels can only be acquired with a frame rate of 0.84 Hz with a similar signal-to-noise ratio. Under these conditions and contrary to the usual placement of the CCD-BSI used on the beamline, only half of the scattering pattern could be recorded, as highlighted schematically by the yellow dashed rectangle in Fig. 1 ▸(*b*). An example of a single-shot image acquired for a total exposure time of 14.5 ms is given in Fig. 2 ▸. During such an acquisition, the estimated deposited energy of the FEL pulse was about 22 mJ cm^−2^. The collected image, obtained after subtraction of a dark background image to remove the beamline stray radiation and the dark level, clearly shows the Bragg peak of the photons magnetically scattered by the magnetization grating structure.

In order to com­pare the detector response linearity to the incoming FEL radiation, the XUV FEL beam intensity was tuned by varying the nitro­gen gas pressure inside the attenuator gas cell chamber placed in the PADReS photon beam transport (Zangrando *et al.*, 2015 ▸). In our experiment, an additional aluminium filter of 780 nm was used to reduce the FEL intensity, preventing any damage to the sample. The incident intensity of the FEL was estimated considering the beamline (∼64%) and Al 780 nm filter (∼21%) transmission, and by recording the energy collected by a calibrated gas intensity monitor, based on the nitro­gen photoionization, placed at the beginning of the photon transport (Zangrando *et al.*, 2015 ▸).

Time-resolved experiments were performed by recording the magnetic scattering patterns for different IR pump fluences, while changing the IR pump to X-ray probe delay. In our experiment, the sample was excited by a short IR laser pulse of 100 fs and probed by a single 60 fs circularly polarized short pulse of FEL XUV rays of 58 eV (Co *M*-edge), both working at 50 Hz. Since the IR laser pulse is derived directly by the same master oscillator seeding the FERMI FEL, the intrinsic arrival time jitter at the sample plane between the two pulses is below 10 fs (Danailov *et al.*, 2014 ▸). Hence, it is possible to perform time-resolved experiments without the need to sort the data in postprocessing using quite com­plicated additional information coming from timing tools along the beam path (Harmand *et al.*, 2013 ▸; Bionta *et al.*, 2014 ▸). This peculiar feature of the FERMI seeded FEL source allows pump and probe experiments to be performed on the fly, where the arrival time between the FEL and the IR laser is varied continuously during the acquisition, moving the mech­an­ical optical delay line of the beamline and collecting the data at the source repetition rate. This reduces sub­stan­ti­ally the acquisition time with respect to the classical approach where the delay between the two beams is varied in a finite number of steps and the data are collected in between two successive motions of the delay line.

**Figure 3:**
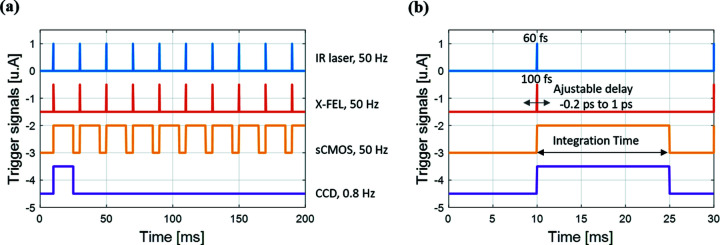
Timing diagram, at larger (*a*) and smaller (*b*) time scales, of the image acquisition signals. Every signal is synchronized with the FEL. The delay of the IR pump pulse is adjustable. Every diffraction peak produced by a FEL probe pulse can be collected with the CMOS camera, whereas only 1 pulse out of 63 can be recorded with a conventional CCD camera.

It is worth noting that even if the pointing of the IR laser beam at the sample plane is controlled by a piezoelectric feedback system (Capotondi *et al.*, 2015 ▸), during our experiment we kept the laser spot size (520 µm × 480 µm) always bigger than the X-ray probe area (370 µm × 350 µm) in order to uniformly pump the sample and minimize the effects of spatial drift between the two radiations during the continuous scan of the delay line. Beam areas are specified with 4σ cuts of the tails of the Gaussian. The acquisition scheme used for every measurement described in the rest of this article is given in the oscillogram presented in Fig. 3 ▸.

Finally, for practical reasons, during this test experiment, the sensor was not cooled using the available Peltier element, but stabilized at 6 °C by water cooling. Nevertheless, due to the intrinsic impulsive structure of the FEL source and the short acquisition time (<15 ms), the background dark signal noise level is relatively low. The subtraction of a reference background image, acquired without illuminating the sample with FEL radiation, removes most of the offset and, under this condition, we estimate a detector noise of ∼4 ADU bins in RMS for a 14.5 ms exposure time.

## 3. Experimental results

### 3.1. CMOS camera linearity at a FEL

The AXIS-SXR linearity has been evaluated using a wide range of FEL fluences, covering a variation of two orders of magnitude. As described previously, the magnetic scattering intensity has been measured as a function of the FEL intensity. In order to safely test the linearity of the CMOS, the FEL intensity has been kept far from the damage threshold of the sensor based on our previous X-ray characterization (Desjardins *et al.*, 2020 ▸).

**Figure 4:**
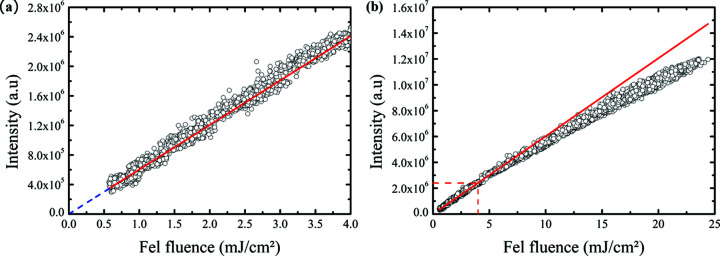
Total signal on the CMOS plotted with respect to the incoming FEL intensity I0 for low fluences (*a*) and for the full range of fluences (*b*). The red curve corresponds to a linear fit of the data points in the low fluence part.

Thus, only a variation in the incoming intensity or a degradation of the sample could explain the variation of scattered intensity. The intensity recorded with the CMOS corresponds to the sum of the pixels over a region of inter­est (ROI) covering the diffraction peak. As shown in Fig. 4 ▸(*a*), we obtain a good linear relationship with a minor offset between the CMOS signal and the incoming X-ray intensity within a variation of one order of magnitude from the minimum of the incident flux, being the incident X-ray flux (I0) monitored as explained above.

The dispersion of the points for each fluence is relatively low (less than 10% around the average), confirming the excellent stability in shot-to-shot power of the seeded FEL. We fit the experimental data by a linear function and conclude that for this fluence range the detector is perfectly linear. As highlighted by the blue dashed line in Fig. 4 ▸(*a*), we have a remaining electric background signal that is not subtracted during our data analysis, which represents less than 50% of the scattered intensity for the lowest X-ray intensity accessible during our experiment.

As displayed in Fig. 4 ▸(*b*), for higher FEL intensities a clear deviation from linearity is observed. This drift does not come from to the CMOS pixel saturation over the considered ROI because the maximum pixel intensity on the image for the highest fluence did not exceed 800 ADU/pixel, while the saturation of the CMOS is much higher, around 65000 ADU/pixel. This particular result was obtained several times on different parts of the sample. The decrease in the response has already been observed on similar samples (Wang *et al.*, 2012 ▸; Philippi-Kobs *et al.*, 2021 ▸) and can be inter­preted simply as X-ray FEL-induced changes of the electronic structure of the material due to X-ray absorption when the FEL fluence reaches levels com­parable to the usual optical laser fluence used in the demagnetization process (Müller *et al.*, 2013 ▸).

### 3.2. FEL CMOS camera pump–probe result

In the pump–probe experiments, we followed the time variation of the intensity of the magnetic scattering, *I*_M_(*t*), after optical pumping by an IR femtosecond laser. Thanks to the stability of the seed FEL source, the variation in intensity from shot-to-shot of the incoming intensity can be considered small and we can therefore use the I0 signal to simply normalize the scattering intensity.

**Figure 5:**
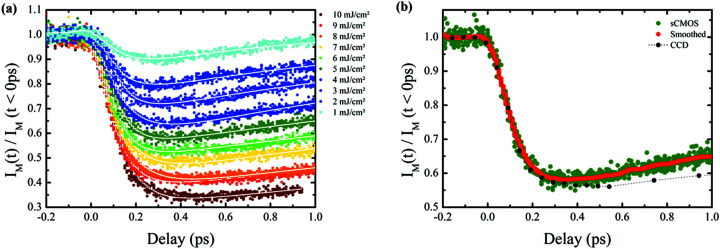
(*a*) Ultrafast demagnetization curves for different pump laser fluences with their respective fit curves in white. (*b*) Curves taken with the CMOS and CCD camera with laser fluences around 5 and 6.9 mJ cm^−2^, respectively. The red line is a smoothed curve corresponding to the CMOS data (see text for details).

Multiple ultrafast demagnetization curves have been per­formed with different laser fluences and the results are pre­sented in Fig. 5 ▸(*a*), where each point corresponds to a single-shot exposure.

The different demagnetization curves for laser fluences ranging from 1 to 10 mJ cm^−2^ are easily distinguishable, demon­strating the feasibility of single-shot pump–probe experiments with the CMOS camera. During our data analysis, we followed the same procedure described elsewhere (Léveillé *et al.*, 2021 ▸).

Each delay scan contains approximately 1000 data points and has been recorded in a total acquisition time of 20 s. By com­parison, in Fig. 5 ▸(*b*), we display a similar delay scan recorded during a previous experiment on the same sample with the standard CCD-BSI camera that needs a total acquisition time of around 600 s (600 shots/point). As can be seen and highlighted by a smoothing of the raw CMOS data (50 neighbouring points), both curves display similar trends, despite the fact that for the CMOS we have one shot per point, while each point corresponds to 600 shots for the CCD-BSI. The small difference after 0.5 ps comes from the slightly different IR laser fluence used to pump the sample in both experiments (5 mJ cm^−2^ for the CMOS and 6.9 mJ cm^−2^ for the CCD-BSI). This discrepancy could be due to a different energy distribution inside the IR pumping laser spot size between the two measurements that were performed with different experimental setups; however, the remarkably identical dynamic on a short time scale (<0.5 ps) com­pared to the short acquisition time shows unequivocally the potential of the CMOS detector in time-resolved scattering experiments. In order to better highlight the data quality, we display as a red line in Fig. 5 ▸(*b*) a moving smooth average, illustrating that we have a similar signal-to-noise between the CMOS and the CCD-BSI. Moreover, it is noteworthy that, as a result of the fastest reading time, the higher data density (recorded points/time inter­val) recorded in the ultrafast decrease during the first few hundred femtoseconds enables a more accurate fit of this part of the curve, which is of tremendous inter­est in the framework of ultrafast demagnetization dynamics. The different fits are performed with two exponential functions convoluted with a Gaussian, instead of the usual three exponential functions used in longer delay experiments (Pfau *et al.*, 2012 ▸). We found a time constant of around 90 fs, with a standard deviation of 13 fs between each fluence, confirming the excellent result obtained using the CMOS. We note that these values are close to those obtained for other Co samples (Yamamoto *et al.*, 2020 ▸; Willems *et al.*, 2020 ▸; Vaskivskyi *et al.*, 2021 ▸). We also found an increasing fast recovery time with respect to the fluence, as was found in previous work (Atxitia *et al.*, 2010 ▸; Von Korff Schmising *et al.*, 2015 ▸).

**Figure 6:**
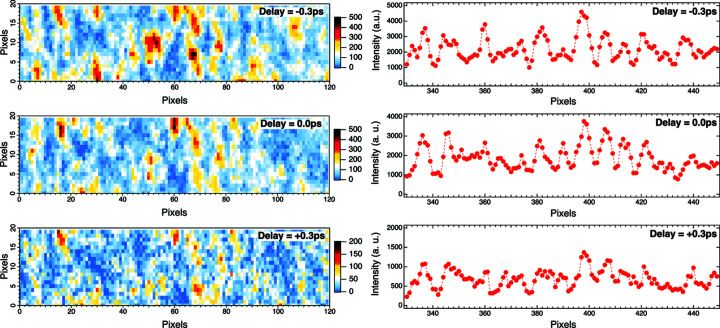
(Left) Zoom on the magnetic Bragg peak intensity for speckles visibility and (right) the associated line plots resulting from the summation over the lines for each row.

So far, we have shown the advantages that the CMOS camera could bring to FEL time-resolved experiments in terms of gain in data acquisition, but we would also like to illustrate that by operating the CMOS camera at 50 Hz, corresponding to the FERMI main frequency, X-ray photon correlation spectroscopy (XPCS) experiments for pump–probe ultrafast dynamics in the ultra-fast time domain can be performed. Until recently, XPCS was widely used for rather slow dynamics processes (order of milliseconds), leaving the local atomic scale behaviour unreachable. However, by splitting the FEL intensity into two beams with an X-ray split and delay line (Osaka *et al.*, 2016 ▸; Roseker *et al.*, 2018 ▸), it is possible to gain access to such dynamics as performed by Shinohara *et al.* (2020 ▸). In order to decorrelate the two signals received by the camera during a single exposure, a speckles contrast analysis needs to be performed. This process requires the recording of the intensities of two split pulses used to calculate the evolution of the specles in time (Gutt *et al.*, 2009 ▸; Dixon & Durian, 2003 ▸). Accessibility to sub-picosecond dynamics could give local information about domain-size-dependent demagnetization processes that was not reasonably accessible with the start-and-stop data acquisition mode, due to the long acquisition time. The magnetic speckles study in XPCS gives information on the variation of the sample area illuminated because it depends on the coherent scattered light inter­ference arriving on each pixel. Thus, it can be used as a marker to qu­antify non­reproducible effects on this area com­pared to the first image. By acquiring multiple images in pump-and-probe mode using the same delay time, it averages and removes those speckles, losing the local information in the process. In Fig. 6 ▸(*a*), we show an example of the speckles visible inside the diffraction peak. We also display in Fig. 6 ▸(*b*) the changes of the magnetic speckles for different time delays (corresponding to a summation over each line for one row). This clearly demonstrates the feasibility of XPCS experiments on magnetic samples at FERMI using the AXIS-SXR camera.

## 4. Conclusion and perspectives

We demonstrated the performance of the scientific back-side-illuminated CMOS for XUV and soft X-ray FEL applications. Even if not yet fully optimized for XUV with a poor detection efficiency, we installed the AXIS-SXR detector inside the vacuum chamber of the DiProI end-station at FERMI FEL. The software detector integration was efficiently achieved using the collaborative Lima tango device. The detector was installed in a vacuum environment and synchronized with the FEL source. We characterized the linear response of the CMOS-BSI for a range of incoming intensities varying by two orders of magnitude and showed that the signal-to-noise ratio is acceptable for FEL applications. We found a good linear response at low fluence, while linearity measurements at higher fluences were affected by the experimental setup. In order to be synchronized with the FEL and pump IR pulses, both operating at 50 Hz, we used a 700 × 700 pixels region of inter­est, which allows a frame rate of 50 Hz. We repeated the experiment for different pump fluences and obtained results of excellent quality. Compared to the usual CCD-BSI camera, we gained a factor of 30 in total acquisition time, with significantly increased statistics, allowing for a more precise analysis of the experimental data. In addition, we can record ‘for free’ single-shot scattering patterns, opening the way for performing ultra-fast XPCS.

On a short-term perspective, with a com­plete integration of the camera in the data acquisition system of FERMI, even an additional gain is expected in the data acquisition time and, by cooling the camera to the lowest temperature, an even better signal-to-noise ratio can be obtained. Furthermore, the new generation of GSENSE400 sensors (Harada *et al.*, 2020 ▸; https://www.gpixel.com) could be easily integrated without any development to increase the efficiency at FERMI FEL working energies or to protect the chip from radiation effect.

On a longer perspective, these new CMOS sensors are under permanent development triggered by scientific applications, such as astronomy (https://www.teledyne-e2v.com/), with bigger and faster sensors or very high efficiency sensors for biology (very low noise CMOS-BSI equipping the Hama­matsu CMOS camera). They will allow a kHz frame rate to be reached (by cropping the sensor in one direction) and will therefore pave the way for many applications in the domain of X-ray scattering and spectroscopy at laser-based X-rays sources, such as, for example, the High Harmonic Generation source (Vodungbo12), or attosecond X-ray spectroscopy (Géneaux *et al.*, 2021 ▸). The integration of a small-pixel CMOS-BSI sensor of 60 mm^2^ reaching a frame rate of 26 Hz is already planned in a collaboration between SOLEIL and Axis Photonique. A larger detector could also be obtained by tiling multichips or dedicated production from the CMOS manufacturer. Although other 2D detectors operating in the MHz regime (https://www.xfel.eu/news_and_events/news/index_eng.html?openDirectAnchor=1701) are already mounted at EuXFEL and have already been used with success (Zhou Hagström *et al.*, 2022 ▸), the difference in price and ease of use between these two categories of detectors promises a long and successful future for CMOS-based 2D soft X-ray detectors.
