# Single-shot experiments at the soft X-FEL FERMI using a back-side-illuminated scientific CMOS detector. Corrigendum

**DOI:** 10.1107/S1600577522001370

**Published:** 2022-02-15

**Authors:** Cyril Léveillé, Kewin Desjardins, Horia Popescu, Boris Vodungbo, Marcel Hennes, Renaud Delaunay, Emmanuelle Jal, Dario De Angelis, Matteo Pancaldi, Emanuele Pedersoli, Flavio Capotondi, Nicolas Jaouen

**Affiliations:** a Synchrotron SOLEIL, L’Orme des Merisiers, Saint-Aubin, BP48, 91192 Gif-sur-Yvette, France; b Sorbonne Université, CNRS, Laboratoire de Chimie Physique-Matière et Rayonnement, LCPMR, 75005 Paris, France; c Elettra-Sincrotrone Trieste, Basovizza, Trieste 34149, Italy

**Keywords:** CMOS, soft X-ray FEL applications, single-shot experiment, time resolved

## Abstract

The name of one of the authors in the article by Léveillé *et al.* [(2022), *J. Synchrotron Rad.* **29**, 103–110] is corrected.

## Abstract 2

The article by Léveillé *et al.* [(2022), *J. Synchrotron Rad.* **29**, 103–110] is corrected.

In the article by Léveillé *et al.* (2022 ▸) the name of the fourth author was given incorrectly. The correct name is Boris Vodungbo, as given above.
